# Thickness- and quality-controlled fabrication of fluorescence-targeted frozen-hydrated lamellae

**DOI:** 10.1016/j.crmeth.2025.101004

**Published:** 2025-03-24

**Authors:** Daan B. Boltje, Radim Skoupý, Clémence Taisne, Wiel H. Evers, Arjen J. Jakobi, Jacob P. Hoogenboom

**Affiliations:** 1 Department of Imaging Physics, Delft University of Technology, Delft, the Netherlands; 2 Department of Bionanoscience, Kavli Institute of Nanoscience, Delft University of Technology, Delft, the Netherlands; 3 Delmic B.V., Delft, the Netherlands

**Keywords:** Thickness control, Frozen hydrated lamella, Quality control, cryo-ET, cryo-LM-FIB-SEM, cryo-SECOM, CLEM, coincident, fluorescence targeted milling, FIB-SEM

## Abstract

Cryogenic focused ion beam (FIB) milling is essential for fabricating thin lamella-shaped samples out of frozen-hydrated cells for high-resolution structure determination. Structural information can only be resolved at high resolution if the lamella thickness is between 100 and 200 nm. While the lamella fabrication workflow has improved significantly since its conception, quantitative, live feedback on lamella thickness, quality, and biological target inclusion remains lacking. Using coincident light microscopy integrated into the FIB scanning electron microscope (FIB-SEM), we present three strategies that enable accurate, live control during lamella fabrication. First, we combine four-dimensional (4D) STEM with fluorescence microscopy (FM) targeting to determine lamella thickness. Second, with reflected light microscopy (RLM), we screen target sites for ice contamination and monitor lamella thickness and protective Pt coating integrity during FIB milling. Third, we exploit thin-film interference for fine-grained feedback on thickness uniformity below 500 nm. Finally, we present a fluorescence-targeted, quality-controlled workflow for frozen-hydrated lamellae, benchmarked with excellent agreement with energy-filtered transmission electron microscopy (EFTEM) measurements and tomograms from electron cryotomography.

## Highlights

• Workflow for lamella preparation in a tri-coincident, fluorescence-integrated FIB-SEM
• Automated fluorescence-targeted preparation of frozen-hydrated lamella
• Live feedback on lamella quality and thickness by three orthogonal methods
• Accurate fluorescence targeting by correction of axial scaling effects

## Motivation

The composition, conformation, and function of most macromolecular complexes depend on their cellular context and must be studied inside cells. Few cells are sufficiently thin to permit direct imaging with cryoelectron microscopy (cryo-EM). Focused ion beam milling enables cryo-EM to visualize macromolecules in cells at high resolution by generating thin sections of frozen-hydrated cells. We show how thin cellular sections can be prepared in a controlled fashion using an integrated light microscope coincident with electron and ion beams. The procedure provides live feedback on the thickness and uniformity of the prepared lamella, reducing complexity and increasing the success rate. Combined with its ability for fluorescence-based targeting, our procedure paves the way toward an automated workflow that allows for control over lamella quality, thickness, and target inclusion, facilitating the routine fabrication of frozen-hydrated cellular sections.

## Graphical abstract

**Figure:**
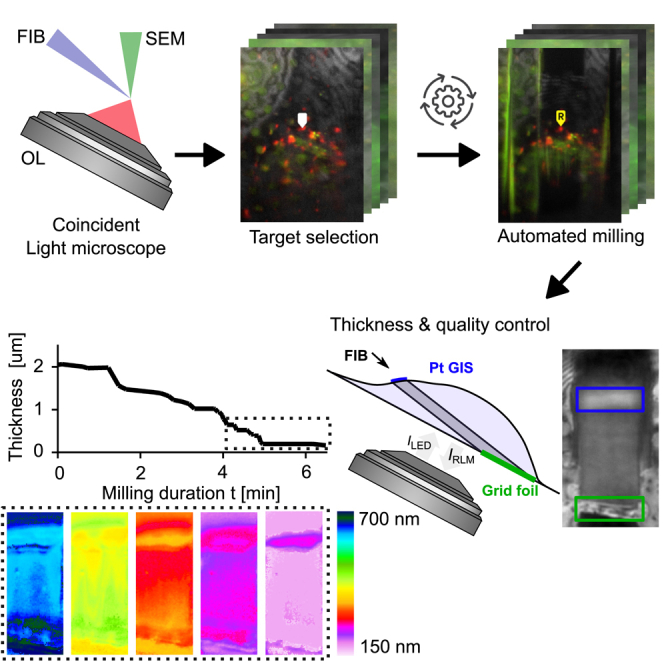

## Teaser

Boltje et al. demonstrate a workflow to prepare thin cellular sections in a thickness-controlled fashion using an integrated light microscope coincident with electron and ion beams. This reduces complexity and increases the success rate, and combined with the ability for fluorescence-based targeting, it paves the way toward a fully automated workflow.

## Introduction

Cryoelectron tomography (cryo-ET) has become an integral technique in the quest for a mechanistic understanding of complex biological processes at the molecular scale, as it remains the sole imaging method capable of discerning intricate structural features within a cell without labeling.^1^^,^^2^^,^^3^ However, its utility in gaining biological insights is often hampered by constraints in sample preparation, particularly the need for thin, ideally artifact-free, cellular sections. These thin sections can be fabricated out of frozen-hydrated cells using a focused ion beam scanning electron microscope (FIB-SEM). In the FIB-SEM, grazing-incidence ion bombardment locally removes cellular material, revealing a cross-section of the cell’s interior (lamella), which is then primed for high-resolution imaging by transmission electron microscopy (TEM).^4^^,^^5^^,^^6^^,^^7^ To achieve optimal structural resolution, the ideal lamella thickness must be around 100–200 nm, remaining well below the inelastic mean free path of electrons in vitreous ice, which is about 320 nm for TEM imaging at 300 keV.^8^

In recent years, various improvements and refinements have been made to the cryo-FIB milling workflow, enhancing throughput, reliability, sample yield, and quality. Gas injection systems (GISs) in the cryo-FIB microscope enable the deposition of a Pt layer, which protects the target region from ion exposure. Maintaining the integrity of this Pt layer during milling is crucial for obtaining thin lamellae and preventing ion-induced damage. Different approaches to *in situ* fluorescence imaging have been integrated into the cryo-FIB workflow to aid in selecting target cells and identifying regions of interest for milling.^9^^,^^10^^,^^11^^,^^12^^,^^13^^,^^14^^,^^15^^,^^16^^,^^17^^,^^18^^,^^19^ However, precise localization of the target in cryo-FIB coordinates remains challenging due to registration errors and aberrations that result from refractive index mismatches (RIMs) during fluorescence microscopy.^20^ *In situ* feedback during cryo-FIB milling on lamella quality—specifically its thickness and uniformity, the condition of the protective Pt layer, and the inclusion of the biological target—could significantly improve the fabrication yield in the frozen-hydrated lamella workflow.

The lamella thickness and uniformity can be assessed using a SEM.^21^^,^^22^ However, this method assumes the lamella is composed of a homogeneous material, which is not the case for cellular samples. Additionally, most methods require independent calibration before each imaging session and come with practical drawbacks.^23^ Quantitative thickness estimations for cellular specimens have been reported when imaging the periphery of a cell in the TEM,^24^ but these have not been extended to the lamella fabrication workflow. Alternatively, using quantitative four-dimensional (4D) STEM (q4STEM) imaging, we recently demonstrated that lamella thickness can be robustly estimated directly in the FIB-SEM without additional calibrations, provided the instrument setup allows for transmission imaging.^23^

Up to now, routine fabrication of sufficiently thin frozen-hydrated sections has remained challenging due to a lack of direct, quantitative feedback on all key parameters that determine the lamella quality: thickness, uniformity, ice contamination, state of the protective Pt (GIS) layer, and inclusion of the biological target.

Here, we utilize a coincident fluorescence microscopy (FM)-FIB-SEM setup^17^ for automated *fluorescence-targeted* lamella preparation and present three complementary techniques to estimate the thickness of a frozen-hydrated lamella during the milling process without requiring prior calibration. We (1) apply the previously presented q4STEM method to frozen-hydrated lamellae and benchmark the measured thickness against energy-filtered transmission electron microscopy (EFTEM), (2) image the lamella from the foil side using reflected light microscopy (RLM) and estimate its thickness based on the known milling geometry, and (3) exploit thin-film interference (TFI) on a per-pixel basis, yielding a fine-grained thickness map of the lamella and providing quantitative feedback on lateral thickness variations. Furthermore, we address the axial scaling effects caused by the RIM during the selection of fluorescence microscopy targets and incorporate this correction into our approach for automated milling of fluorescent targets. Finally, we integrate all of these techniques to show a fluorescence-targeted lamella fabrication workflow with precise control over both lamella thickness and quality.

## Results

### Fluorescent targeting under RIM

In our three-beam coincident setup, cryofluorescence microscopy is used to identify targets in frozen-hydrated cells, after which the surrounding material is ablated with the FIB to create a lamella containing the target. Due to the large temperature difference between the optical objective (at room temperature) and the sample held at 100 K, a dry objective lens (OL) $\left(n_{1}=1.0\right)$ is used to image the cellular material, which primarily consists of vitrified water ($n_{2}=1.28$ at T=109K).^25^ This creates a RIM, resulting in a depth-dependent deformation of the optical microscope’s axial coordinate.^20^ Correctly accounting for the RIM-induced axial deformation is crucial when fabricating lamellae around fluorescent targets embedded in frozen-hydrated cells.

**Figure 1:**
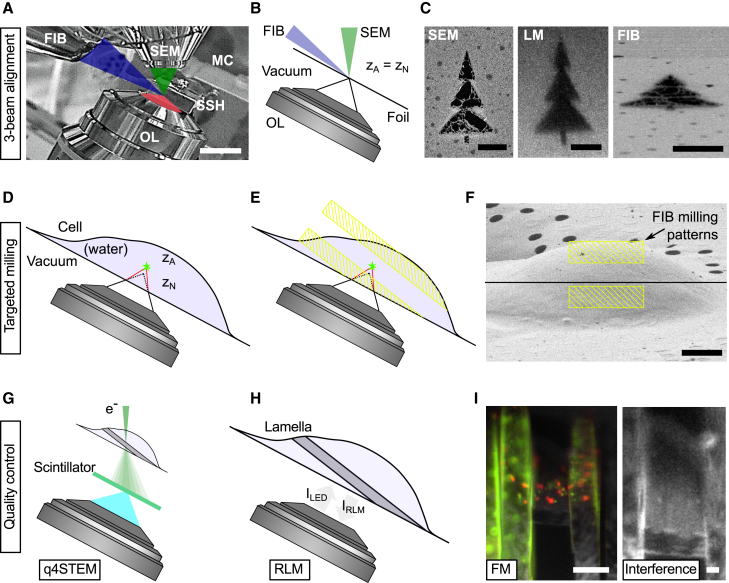
Cryogenic, coincident fluorescence, electron, and ion beam microscope used for alignment, targeted milling, and quality control The alignment of the experimental setup used for lamella fabrication (A–C), the targeting and milling approach (D–F), and techniques for quality control (G–I). (A) Infrared photograph acquired with the camera mounted on the FIB-SEM host system showing the three coincident beams (SEM, LM, and FIB) along with the microcooler and objective lens (OL) mounted inside the microscope chamber. The EM grid containing frozen cells is contained in the sample shuttle holder (SSH), and epi-fluorescence imaging is done from the bottom (transparent red). (B) Alignment of the optical microscope is done by moving the OL, without a RIM present. The AutoGrid and coverslip have been omitted for clarity. (C) Milled pattern imaged with three imaging modalities after alignment; the light microscopy image is acquired by collecting reflected light from the sample. (D) When targeting fluorescence inside a cell, the axial coordinate is distorted due to RIM. The measured axial position $\left(z_{N}\right)$ of the fluorescent emitter does not coincide with the actual position $\left(z_{A}\right)$. (E) The bottom milling pattern is positioned close to the coincident point, as the fluorescent emitter *always* sits further away from the OL due to axial scaling. (F) Placement of the asymmetric milling pattern with respect to coincidence (black line) in the FIB field of view prior to rough milling a cellular structure. (G) Schematic diagram showing the geometry and detection principle used to measure lamella thickness with q4STEM.^23^ By collecting scintillation light from the scattered electron pattern with the optical system, the lamella thickness can be determined. (H) Schematic showing the lamella orientation with respect to the OL. The lamella thickness can be estimated by collecting the reflected light intensity from the lamella $\left(I_{\text{RLM}}\right)$, both through the known imaging geometry and interference effects. See also (I), right. (I) The fluorescence intensity can be monitored during and in between milling; thus, the presence of the biological target inside the lamella is guaranteed. Thin-film interference effects are present in RLM, as seen by the intensity gradient going from the bottom (dark) to the top (bright) of the lamella. In addition, interference fringes are visible, originating from a Newton interferometer geometry. Both of these effects can be exploited to determine the thickness of the lamella. Scale bars: (A) 2 cm, (C) 5 μm, (F) 5 μm, (I) 10 μm (left), and 2 μm (right).

To compensate for the axial compression caused by the RIM, the z position of the interface between the vacuum and specimen must be precisely determined. In our coincident three-beam microscope, the sample holder is oriented such that an incident angle of 10° is maintained with the FIB, and consequently, the SEM has an incident angle of 118°. The optical objective is positioned below the specimen and images the region of interest (ROI) at right angles through the electron microscopy (EM) grid, as shown in Figure 1A.

When an AutoGrid-mounted sample is loaded into the system, the correct height for three-beam coincidence is initially achieved by moving the OL to the coincident point of the FIB-SEM. To identify the z position of the air-specimen interface, the objective stage is used to focus on the grid foil while avoiding ice in the optical path, thereby preventing a RIM. This ensures that the measured axial (microscope or nominal, $z_{N}$) position of the grid foil matches the actual position $z_{A}$ (Figure 1B). The alignment pattern is then imaged using a SEM, FIB, and light microscope (LM), with the light microscopy image captured by collecting reflected light from the sample (Figure 1C).

With a numerical aperture (NA) of 0.85 and the refractive indices of both the OL and the sample, the axial scaling behaves linearly as long as fluorescent emitters are no deeper than about 9 μm.^20^ The upper thickness limit for obtaining vitreous plunge-frozen-hydrated cells is 10 μm.^26^ Therefore, the focal shift (i.e., the difference between the actual and measured emitter positions) for a targeted emitter ranges from 0 to ∼2.7 μm, scaled linearly at an ∼300 nm shift per micrometer of emitter depth. This is schematically illustrated in Figure 1D, where a fluorescent object at actual depth $z_{A}$ is imaged at the axial coordinate $z_{N}$ due to the optical microscope’s aberrations.^20^

To quantitatively correct the focus shift, precise measurement of the scaled distance is needed, which is achieved by determining the focal length difference between the foil (the RIM interface) and the fluorescent emitter. Since lamella fabrication occurs in a stepwise process, with lamella thickness after rough milling typically ranging around 2 to 3 μm, we adopted an alternative approach. When imaging from a low $\left(n_{1}=1\right)$ to a high $\left(n_{2}=1.28\right)$ refractive index, the optical microscope’s axial coordinate system appears compressed. This enables an asymmetric milling pattern where the bottom pattern is placed a few hundred nanometers away from the coincident point (Figures 1E and 1F). Consequently, the top milling pattern is set ∼2.7 μm away from the coincident point, ensuring that the fluorescent ROI is captured within the rough-cut lamella.

Quality and thickness control are ensured through three complementary techniques that estimate the thickness of a frozen-hydrated lamella during the milling process. We apply our previously presented q4STEM method^23^ to frozen-hydrated lamellae (Figure 1G). By leveraging the known imaging geometry and interference effects present in the RLM, we can estimate lamella thickness and verify the presence of the fluorescent target inside the lamella using FM (see Figures 1H and 1I). Further details on these methods are discussed below.

### Automated milling of fluorescent targets

**Figure 2:**
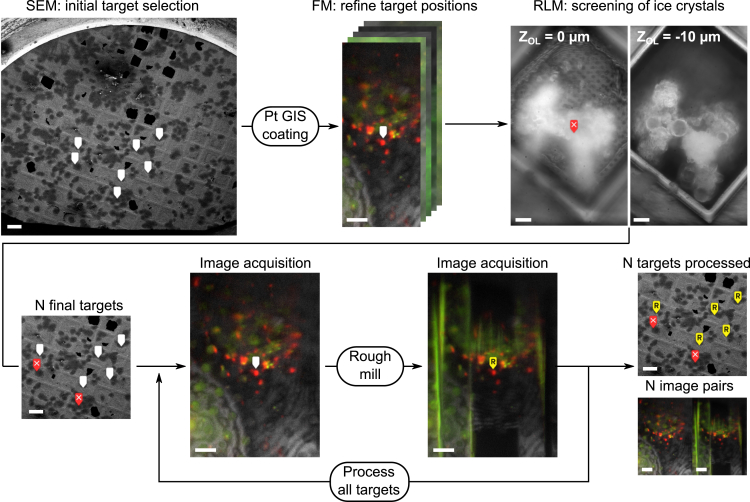
Fluorescence targeted, automated lamella preparation workflow for selected cellular targets Initial target cells are identified in a low-magnification SEM image (white markers, 1 keV, 25 pA, ∼2mm horizontal field width). After applying the Pt coating, the target positions are refined through fluorescence imaging, and targets are screened for the presence of ice crystals using the RLM. n final targets are rough milled automatically while acquiring FM images before and after. Scale bars: 100 μm (SEM images), 10 μm (RLM images), and 100 μm (FM images).

We set out to implement *in situ* fluorescence targeting to establish automated lamella preparation on selected cellular targets. Our workflow is illustrated in Figure 2, where potential target cells are identified using a low-magnification SEM image (white markers) prior to applying the Pt coating with the GIS. Each site is imaged using an FM to refine target positions, and additionally, the sites are screened using RLM. During the sample preparation, ice crystallites occasionally form, and larger clusters can obscure the target site. These clusters are often found below the foil side of the grid and thus remain invisible in the SEM image. They not only obstruct fluorescence imaging but also induce additional axial scaling when located directly beneath the target. If present along the lamella’s milling direction, they may also hinder subsequent tilt series acquisition in the TEM. RLM imaging using the integrated optical microscope helps identify and discard these sites (Figure 2, red marker with white cross), thereby increasing the success rate of high-quality lamella for cryo-ET.

Once n final milling targets have been selected, asymmetric rough milling of the lamella is performed in an automated fashion using the stored xyz stage coordinates obtained from FM imaging. For each milling site, the Odemis acquisition software positions the stage and automatically captures images in the relevant channels of the LM.^27^ This imaging occurs both before and after rough milling, resulting in n milled sites (yellow marker with an R in Figure 2) and corresponding image pairs. More examples of these image pairs are shown in Figures S2 and S3 and were acquired over ∼4 weeks during the milling of different biological specimens.

In this study, the Helios Nanolab 650 FIB-SEM (Thermo Fisher Scientific) was used to carry out the milling, with our integrated cryo-LM retrofitted as described previously.^17^ Instructions to mill a pre-defined pattern with the FIB are sent through the XTLib interface (Thermo Fisher Scientific) by adjusting the SEM scan rotation. An iFast script (Thermo Fisher Scientific) executes the milling process, after which the scan rotation is returned to its original value, indicating that milling at this site is complete. Similar strategies for controlling beam, stage, and imaging operations can be employed through the instrument API of other manufacturers. The total time to perform stress-relief cuts, mill an initial lamella, and acquire the LM images is ∼4–5 min per site. The rough lamella can then be thinned down to <200 nm, with quantitative feedback on the thickness, as outlined below.

### Thickness determination through q4STEM

**Figure 3:**
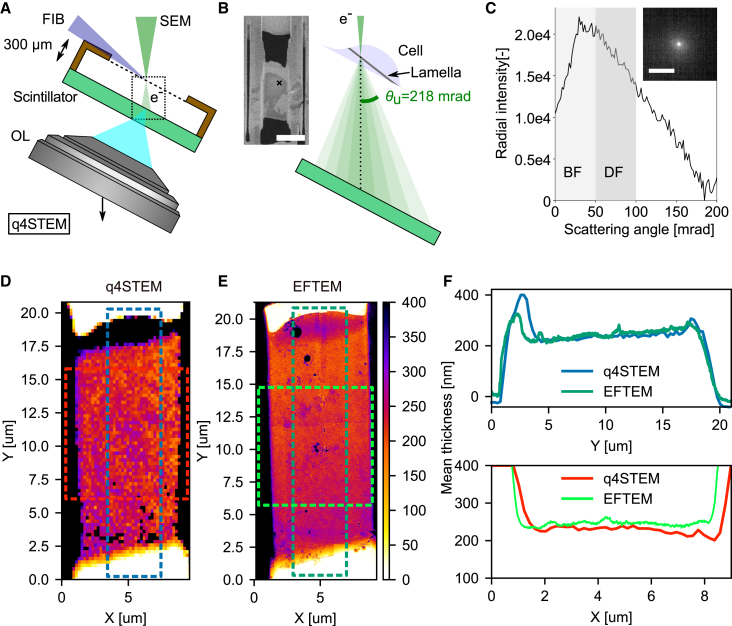
q4STEM-measured thickness of frozen-hydrated lamella compared quantitatively with thickness estimates from energy-filtered TEM (A) Schematic diagram showing the geometry and detection principle used to measure lamella thickness with q4STEM. The OL focus is moved downward (arrow, along the electron optical axis) to the top scintillator surface, where the scattered electron pattern is visualized by collecting the scintillation light (transparent blue). (B) Schematic illustration showing the scattering process in more detail (dotted box in A). The left inset shows a SEM image from an intermediate milling step; the black cross marks the position at which the q4STEM measurement was acquired. (C) The integrated radial intensity profile as computed from the acquired image (see inset). The thickness is determined by taking the ratio of the virtual dark- and bright-field intensity sum, as discussed in earlier work.^23^ (D) q4STEM thickness map after correcting for the SEM angle of incidence of 52°. The map consists of a 64 × 100 pixel grid acquired with a 200 nm pixel size. (E) EFTEM thickness map from zero-loss imaging after correcting the lamella pre-tilt of 10°; pixel size is 3.59 nm. A baseline correction has been applied to the EFTEM data to yield zero thickness outside the lamella (vacuum). (F) Mean line profiles along the x and y directions of the lamella. The dotted boxes in (D) and (E) annotate the region across which the mean intensity was computed. Scale bars: (B) 10 μm and (C) 33 μm or 109 mrad.

Reproducible FIB micromachining of high-quality frozen-hydrated lamella requires robust control over the sample thickness during the milling process. The scattering patterns of transmitted electrons can be recorded by converting our integrated LM into an optical 2D STEM detector. As shown in previous work, this is achieved by replacing the indium tin oxide (ITO)-coated glass coverslip of the integrated optical microscope with a scintillator positioned directly below the AutoGrid (Figure 3A).^17^^,^^23^ While we previously used a yttrium aluminum garnet (YAG) scintillator in our proof-of-principle work, its optical emission spectrum in the visible range conflicts with the absorption and emission spectra of typical fluorescent targets. For this reason, we opted for lutetium aluminum perovskite (LuAP), which has a relatively low optical emission wavelength and interferes only with 440 nm-centered fluorescence emission. To record electron scattering patterns, the OL is lowered along the SEM optical axis to focus on the top scintillator surface while maintaining alignment with the SEM (Figure 3A). When the estimated lamella thickness is below ∼1 μm, the thickness can be determined using q4STEM.^23^ With the SEM in spot mode, the optical microscope focus is fine-tuned through direct electron beam exposure on empty grid foil holes (Figure 3B, inset). Individual scattering profiles from different parts of the lamella are then recorded using the SEM beam shift. In our setup, temporal coordination of electron exposure is managed by controlling the fast beam blanker on our Helios Nanolab 650 (Thermo Fisher Scientific) via the transistor-transistor-logic (TTL) signal output from our optical camera. At each probe position, the lamella thickness is calculated through a radial sum over the scattering profile (Figure 3C) and computing the integrated dark-field/bright-field (DF/BF) ratio. The DF/BF ratio, along with an estimate of the most common scattering angle, can then be compared to tabulated data from Monte Carlo (MC) electron scattering simulations to derive the final thickness.^23^

To validate the robustness of thickness estimation using q4STEM on frozen-hydrated lamella, we employed fluorescence-guided FIB milling to fabricate a lamella from vitrified HeLa cells stably expressing mRFP-EGFP tandem fluorescent-tagged microtubule-associated protein 1A/1B light chain 3B (mRFP-GFP-LC3), a dual fluorophore probe used for studying autophagosomes and their trafficking^28^ (Figure S4). After automatic milling to an approximate thickness of 250 nm, we acquired a 2D convergent beam electron diffraction pattern at each pixel position of a 2D STEM raster covering the lamella. The resulting 4D STEM dataset was then evaluated using the q4STEM method, yielding local thickness measurements at each probe position, corrected for the non-perpendicular incidence of the electron beam (Figure 3D). We then transferred the sample to a JEOL JEM3200-FSC TEM operating at 300 kV and acquired a baseline-corrected EFTEM thickness map using zero-loss imaging (Figure 3E). The two techniques showed good agreement, with a mean thickness difference of only 7 nm across the central 4 μm region of the lamella (Figures 3D and 3E).

Figure 3F shows the mean line profiles corresponding to overlays in the respective 2D maps. Both the q4STEM and EFTEM profiles reveal a ∼50 nm reduction in thickness from the Pt-coated leading edge to the trailing edge of the lamella, caused by the diminished incident ion flux along the lamella due to the grazing angle. The bump, indicating an apparent increase in thickness at y=3 μm of the lamella, is attributed to the platinum-rich GIS coating used to protect the lamella during FIB milling, as the scattering profile is interpreted using simulated data of pure frozen water.

We assessed the possibility of beam-induced damage due to low-voltage electron exposure in the SEM by examining a sample of lysosome crystals, as detailed in the supplemental information (Figure S5). Our analysis focused on low-order reflections due to the limited sensitivity of the scintillator-based detection method. With increasing electron fluence, the intensity of the 0-order peak increases due to electron-beam-induced degradation of the crystal structure. While a more detailed assessment of radiolytic damage affecting higher-resolution features would require future studies using a more sensitive, pixelated direct-detection STEM detector, we argue that thickness determination via q4STEM remains a viable method, as we apply q4STEM exclusively to the edges of the lamella, located 2–10μm away from the ROI, for tilt series acquisition to avoid damage to critical areas. To support this, we have performed MC simulations of 30 kV electrons impinging on a 1,000 nm lamella using CASINO.^29^ The MC electron trajectories suggest that 95% of the deposited energy remains within a maximum radius of 50 nm of the incident beam profile, which is consistent with previous estimates.^30^ The cross-sectional diffusive radius of reactive radiolytic products in amorphous water ice, mediated through, for instance, low-energy (secondary) electrons, is reported to range between 2 and 70 nm.^31^^,^^32^ We, therefore, estimate that beam-inflicted radiolytic damage is likely restricted to several tens of nanometers off the q4STEM scan line and will not affect the ROI at several micrometers distance from that scan line.

### Thickness and quality control using RLM

**Figure 4:**
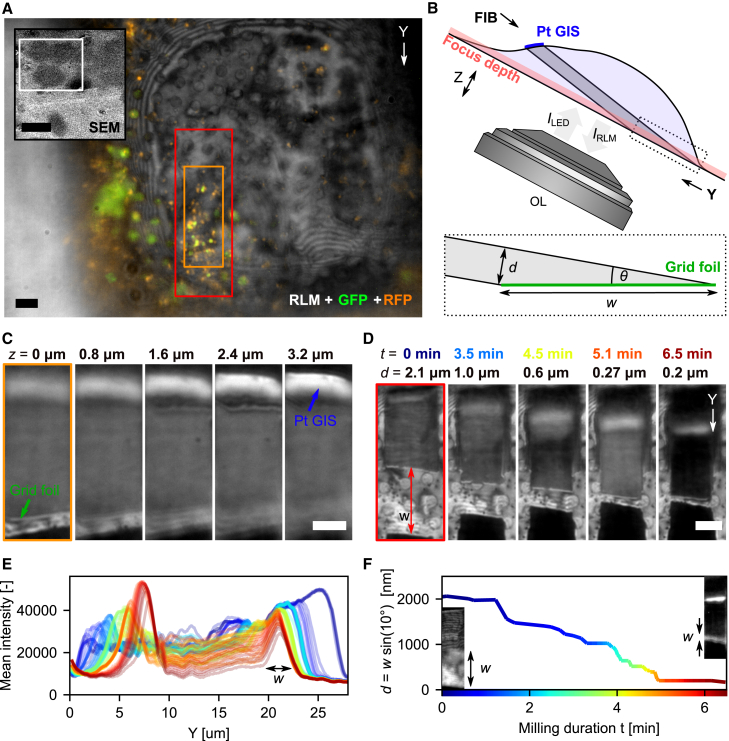
Targeted and quality-controlled FIB milling with *in situ* fluorescence and reflected light imaging (A) Initial target selection in HeLa cells expressing mRFP-GFP-LC3 using fluorescence microscopy (colors) overlaid on reflected light (grayscale). Green and yellow: autophagosomes; red: autophagolysosomes. The light microscope field of view (FOV) is denoted by the white rectangle in the top left inset, showing the grid square in the SEM overview image. (B) Sketch showing the lamella and RLM imaging geometry. The optical focus depth (red translucent are) is about 500 nm. The dotted box shows the lamella geometry close to the grid foil. The lamella thickness d can be estimated by measuring distance w along the tapered wedge of the lamella by d=w·sin(θ). (C) Cropped slices (orange rectangle in A) from an RLM z stack acquired with the lamella milled to about 300 nm in thickness. The top and bottom sides of the lamella can be imaged by changing the optical focus (Pt GIS and foil sides as green and blue annotations, respectively; also visible in B). (D) Various stages (time points) of the lamella milling process (FOV cut to red marked area from A). The lamella thickness d can be estimated by measuring w along the bottom wedge of the lamella. (E) Mean x RLM intensity profiles along the lamella length y (line color indicates milling progression/time according to the color scale in F). w can be measured from the width of the foil-side intensity peak. (F) Estimated lamella thickness d versus milling duration. The two insets show the lamella as imaged in RLM at the start and endpoints of thinning. The lamella thickness can be estimated to ∼250 nm. Scale bars: (A) 5 μm and 50 μm (inset), (C) 5 μm, and (D) 5 μm.

Live feedback on lamella thickness and quality during milling can be obtained by acquiring RLM images with the coincident LM in addition to FM targeting (Figure 4). We demonstrate this process using fluorescence-targeted lamella preparation from HeLa cells expressing mRFP-GFP-LC3, which allows simultaneous localization of autophagosomes and autophagolysosomes. First, ROIs are selected based on the fluorescence images (Figure 4A) of suitable cells, originally chosen via the low-magnification SEM image (Figure 4A, inset). The overlay of the two-channel FM image with the simultaneously acquired RLM image clearly shows the fluorescence embedded in the context of the frozen-hydrated cell and grid support (Figure 4A).

FIB milling is then performed at a 10° angle relative to the plane perpendicular to the LM’s optical axis, creating the geometry shown in Figure 4B. The optical focus depth is ∼500 nm (red shaded area), allowing focus on either the top (Pt, blue) or bottom (foil, green) side of the lamella by adjusting the z position of the sample stage. The lamella thickness is estimated by measuring the width w of the bottom (foil) side of the lamella (Figure 4B, inset). The thickness d is then calculated using the relation d=w·sin(θ), where θ is the milling angle.

Acquiring an RLM z stack during intermediate milling steps allows for assessing the milling process beyond thickness estimation. Both the foil and Pt GIS sides can be brought into focus (Figure 4C), allowing us to evaluate the (1) lamella thickness d by measuring w from the foil side, (2) state and uniformity of the Pt GIS layer, which protects the lamella during ion beam milling, (3) overall uniformity of the milled lamella, and (4) accuracy of the milling angle by measuring the defocus (4 μm) over the full lamella length (22.4 μm), yielding θ=arcsin(4μm/22.4μm)=10.2°. By setting the optical focus between the top and bottom of the lamella (at 1.6 μm in Figure 4C), an approximate lamella thickness d can be estimated, while the highly reflective Pt GIS layer is used to assess the protective layer’s uniformity.

Snapshots showing the progress of the milling process are presented in Figure 4D. These images are taken after pausing FIB milling, with the foil side of the lamella in focus. The width w decreases as the lamella becomes thinner, and the instantaneous thickness d is displayed at the top of the snapshots along with the milling duration and color coded. Additionally, a series of RLM images is acquired during FIB milling, with the optical focus set between the bottom and top of the lamella. The mean reflected intensity along the y axis is plotted in Figure 4E for different milling stages. Characteristic intensity peaks at the beginning (y=∼7 μm) and end (y=∼25to∼20 μm) of each line trace correspond to the bottom and top sides of the lamella, respectively. By measuring the width w at each stage, the lamella thickness can be calculated and plotted against milling duration (Figure 4F). The insets show the first and final images used to determine w. This method enables monitoring of the milling process down to a thickness of around 1 μm.

**Figure 5:**
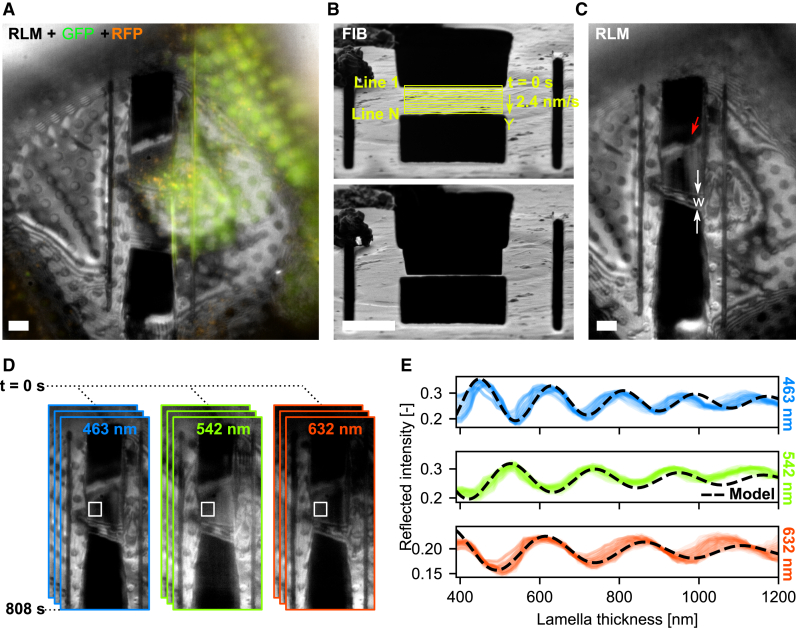
Reflected light intensity monitors lamella thickness during milling (A) Lamella after rough milling as imaged with fluorescence microscopy (colors) overlaid on reflected light (grayscale). At this stage, the lamella thickness d is ∼2.6 μm (w= 15.3 μm). (B) The lamella after rough milling (top) and after thinning (bottom) as imaged with the FIB. The lamella is evenly thinned by defining a cleaning cross-section pattern in the FIB field of view (yellow marked area), having a constant milling rate of 2.4 nm/s along the FIB y direction. The FIB milling progresses line by line at this rate, starting at the top (line 1) and ending at the bottom (line N). (C) Lamella after thinning as imaged with reflected light (grayscale). At this stage, the lamella thickness d is ∼430 nm (w= 2.5 μm), and the Pt GIS layer has been partially removed (red arrow), preventing further thinning. (D) Reflected light images are recorded in three different channels during the even lamella thinning. (E) For each reflected light channel, the normalized reflected intensity is plotted against the lamella thickness. 35 individual intensity traces are plotted for each channel (from white rectangle in D), along with the reflectivity model based on thin-film interference (dashed black, Equation 1). All scale bars: 5 μm.

TFI effects can be leveraged to achieve more accurate lamella thickness determination than with geometric estimations, as demonstrated by Last et al., who utilized multicolor RLM to measure thickness at the periphery of vitrified cells grown on cryo-EM support films.^24^ We demonstrate the application of TFI for thickness determination in our coincident LM by first milling a rough lamella to ∼2.6 μm thickness, as seen in Figure 5A.

Next, a cleaning cross-section pattern was defined within the FIB field of view to thin the lamella at a consistent rate (Figure 5B, top). The milling process proceeded line by line, with an effective rate of 2.4 nm/s along the FIB y axis to ensure even thinning, beginning from the top (t= 0 s) and continuing until a thin lamella remained after completion (Figures 5B, bottom, and 5C). The final thickness, ∼430 nm, was derived from the width w measured in the RLM image. Further thinning was prevented by a discontinuous Pt GIS layer, highlighting the utility of monitoring Pt coating integrity during milling.

We acquired RLM images during FIB milling using three different wavelengths (λ= 463, 542, and 632 nm, 189 images per channel, 567 total images), as depicted in Figure 5D. For each wavelength, 35 individual line traces were extracted from the white rectangles and plotted against lamella thickness. The reduction in thickness was calculated based on the image timestamp and the constant milling rate of 2.4 nm/s. A lamella thickness offset of 390 nm was applied to the traces, which falls within the error margin of the geometric estimate, especially given the non-uniformity of the protective Pt GIS layer. The dashed black line represents the reflectivity model r(d,λ,T), similar to^24^

(Equation 1) $$r\left(d,\lambda ,T\right)=r_{\text{mea}}+\frac{r_{A}e^{-d/L}}{2}\;\cos\left(\frac{4\pi \;d\;n_{2}\left(\lambda ,T\right)}{\lambda }+\pi \right)$$

Here, $r_{\text{mea}}$ and $r_{A}$ are constants adjusted to match the measured reflected intensity, as determined from the acquired images. d is the lamella thickness, and L is a constant accounting for scattering and coherence loss in the thin film, set to 500 nm. The refractive index values for amorphous ice, $n_{2}\left(\lambda ,T\right)$, are taken from Kofman et al*.*^25^

Good agreement between the reflectivity model and the measured data was observed across all three wavelengths, allowing lamella thickness to be determined according to the method outlined by Last et al*.*^24^ To streamline both data acquisition and analysis, we opted to measure reflected intensity for a single wavelength only. By performing error minimization on the reflectivity data, using the geometric thickness estimate as an initial approximation, we can generate 2D thickness maps of the lamella as a function of milling progress, as shown in the next section.

### Controlled lamella fabrication workflow

**Figure 6:**
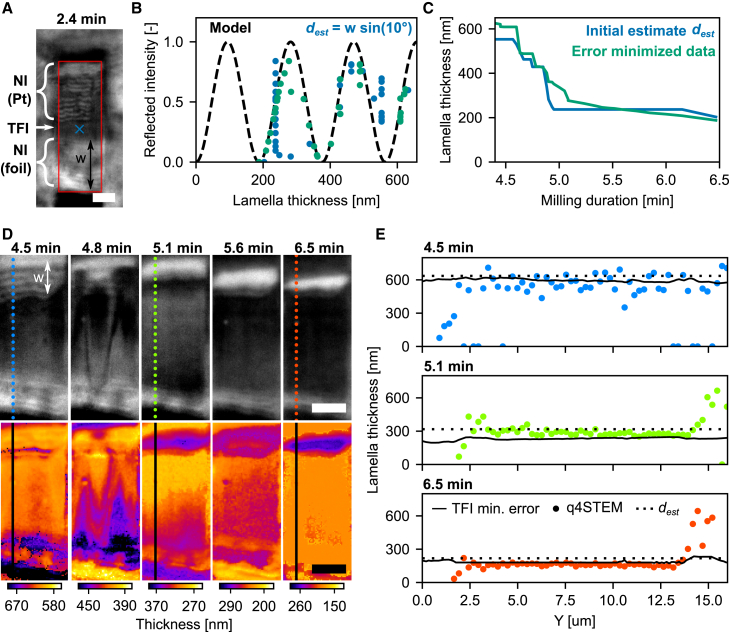
Thickness-controlled lamella fabrication (A) The lamella imaged in RLM (λ= 463 nm) at an intermediate milled state (d≈ 1.5 μm). The bottom (foil) and top (Pt GIS) sides of the lamella are wedge shaped and act as a Newton interferometer (NI). The region between has a more uniform thickness, and hence thin-film interference (TFI) occurs. (B) With milling progression, the reflected intensity is recorded (at the blue cross in A), along with the lamella thickness estimate $d_{\text{est}}=w\;\sin\left(10{}^{\circ}\right)$ from the milling geometry (blue scatter). Error minimization is performed using a normalized version of the reflectivity model (dashed black), which outputs a refined lamella thickness estimate (green scatter) and finally yields (C) the lamella thickness versus milling duration. (D) The lamella imaged in RLM at varying stages of milling (top, grayscale). q4STEM data have been acquired at three time points during the milling (dotted colored lines). The error minimization process as outlined in (B) and (C) can be performed for all pixels in the RLM images, yielding thickness maps at varying stages of milling (bottom, colored). (E) Lamella thickness line traces along the y direction at three different time points. Thickness measurements include q4STEM (colored dots, see also D, top), the error minimization of the normalized reflectivity (black lines, see also D, bottom), and estimates from geometry $d_{\text{est}}$ (dotted black lines). The increase in measured thickness around y = 15 μm is due to the increased density of the Pt GIS layer. All scale bars: 5 μm.

Finally, we combine the above into a single workflow for quality- and thickness-controlled fabrication of fluorescence-targeted lamellae. Initial target selection is done with fluorescence microscopy, and quality control is performed using RLM, as discussed earlier. In reflected light imaging, the wedge-shaped endpoints of the lamella act as a Newton interferometer (NI) due to the changing thickness (Figure 6A). From an approximate lamella thickness of 1.5 μm, a region of constant thickness starts to appear in the middle, where TFI occurs. The normalized reflected intensity is recorded at a single point (blue cross) and plotted using the geometrical thickness estimate $d_{\text{est}}$ in Figure 6B (blue scatter). The thickness can then be further refined through error minimization using the normalized version of the reflectivity model (Equation 1), yielding the green scatter in Figure 6B, and the green curve in Figure 6C. This reflects more intricate thickness differences compared to the initial (geometrical) thickness estimate, partly because the thickness measurements come from different locations on the section but also because the (local) lamella reflectivity is more sensitive to thickness variations.

As reflected light *images* are acquired, this process can be extended to all the pixels on the lamella showing TFI, thus converting a grayscale reflected light image into a 2D thickness map of the lamella, as shown in Figure 6D. To visualize thickness variations at each stage of lamella thinning, the minimum and maximum values of the color scale are adjusted for each image. At three time points during lamella thinning (4.5, 5.1, and 6.5 min), q4STEM data along the y direction were acquired (colored dots). Along with the line traces of the 2D thickness maps (black lines) and the thickness estimate from the milling geometry (dotted line), the lamella thickness is plotted along the y direction in Figure 6E. The three different methods are in good overall agreement, showing approximate lamella thicknesses of 600, 300, and 200 nm at different stages of milling, although $d_{\text{est}}$ overestimates the thickness below 300 nm. The mean differences between q4STEM- and TFI-measured thicknesses are −57, 56, and −21 nm for the three time points, resulting in a relative difference of 10%–20%. At the 6.5 min time point, a lamella thickness of 188 nm was reached, with a uniformity of less than 5 nm (one sigma) from the error-minimized TFI data. Since the protective Pt GIS layer was too thin and non-uniform, polishing was stopped.

**Figure 7:**
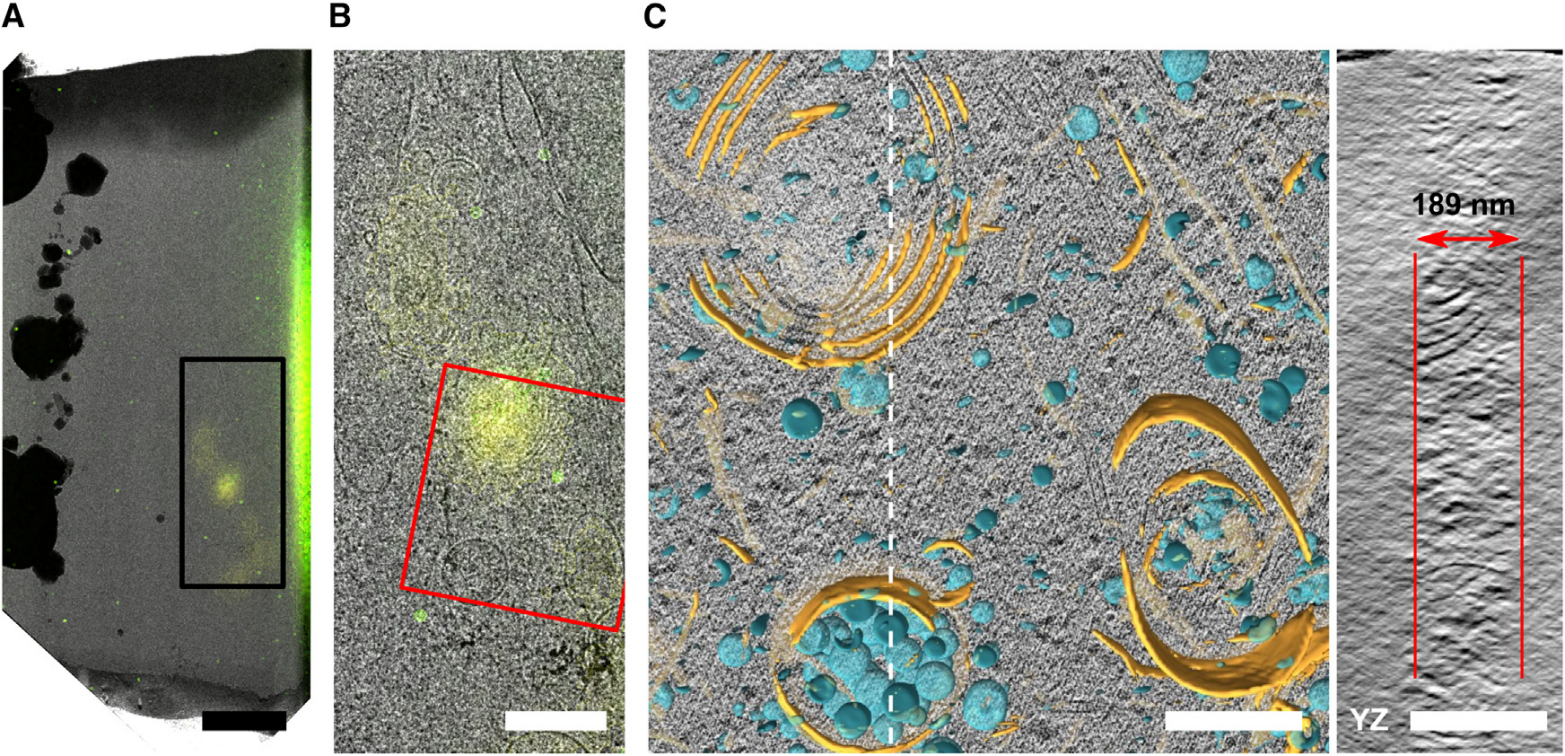
Cryoelectron tomography with fluorescence-targeted, thickness- and quality-controlled FIB-milled lamella (A) Overview image of the lamella in TEM with overlaid fluorescence data. TEM and fluorescence images are aligned based on the reflected light image. This overlay is used for tomography target selection. (B) Zoom of the black boxed area in (A). (C) Snapshot from the reconstructed tomogram with membrane segmentation as an overlay, acquired at the red rectangular area marked in (B). A yz slice taken along the dashed white line is shown on the right, annotating the lamella thickness as measured from the reconstruction. Scale bars: (A) 2.5 μm, (B) 1.0 μm, and (C) 250 nm.

The polished lamella was unloaded from the FIB-SEM using a cryogenic transfer system and loaded into a JEOL JEM3200-FSC TEM operated at 300 keV. An overview image was acquired in the TEM (see Figure 7A). After polishing was completed, fluorescence data were overlaid on the overview image, and alignment between the TEM and the (reflected) light microscopy data was performed using easy cell-correlative light to electron microscopy (eC-CLEM).^33^ This overlay is used to set the ROI for tomogram acquisition (Figure 7B, red box). A snapshot from the acquired tomogram is shown in Figure 7C (left), along with the segmentation overlay. The lamella thickness is measured from the yz slice along the dashed white line (Figure 7C, right), yielding 189 nm, which is in close agreement with the 188 nm estimate from TFI.

## Discussion

We have presented three different methods to accurately determine the lamella thickness during FIB milling using an LM integrated in a FIB-SEM system: (1) by selecting a scintillator better suited for use in an FM, we were able to apply an earlier presented q4STEM technique^23^ to the cryogenic lamella fabrication workflow. Benchmarking this technique to EFTEM showed good agreement in the determined thickness. (2) The lamella thickness was estimated during milling via the known milling and imaging geometry, which produced reliable estimates between 2,000 and 400 nm. (3) Exploiting TFI phenomena on a per-pixel basis yields a fine-grained thickness map of the lamella, thus providing quantitative feedback on the lateral thickness variations. The final lamella thickness estimate was compared with the thickness determined from reconstructed TEM data and showed excellent agreement.

We further showed how to target fluorescent regions of interest while correcting for the axial distortion induced by the inherent presence of a RIM when imaging into a frozen cell. RLM is used to inspect milling sites to discard those sites that have parasitic ice present underneath the EM grid. A rudimentary form of communication between the FIB and FM control personal computers (PCs) is used to perform automated rough milling of fluorescent targets.

For different sample types, such as specimens prepared by the Waffle method or high-pressure frozen (HPF), the thickness can extend up to ∼200 μm.^34^^,^^35^ As such, depth-dependent axial scaling effects need to be considered and quantitatively corrected for by accurately measuring the axial coordinate $z_{N}$ (distance between refractive index interface and emitter) as observed in the optical microscope.

Our methods provide live feedback on lamella thickness and uniformity, the integrity of the Pt coating layer, and the presence of the fluorescent target when preparing a lamella out of a frozen-hydrated cell. This reduces the complexity of the milling process and substantially increases the yield for producing lamella suitable for high-resolution cryo-EM analysis. Combined with its ability for fluorescence-based targeting, our procedure paves the way toward an automated workflow that allows for fluorescence, thickness, and quality control, facilitating the routine fabrication of frozen-hydrated cell sections and highlighting potential advantages of coincident FM-FIB-SEM instruments in workflow automation.

### Limitations of the study

q4STEM and RLM thickness determination during the milling process requires a coincident light microscopy setup integrated in the FIB-SEM system. This is absolutely required for the RLM measurements; for q4STEM, an optional pixelated STEM detector may also be used and could provide improved sensitivity compared to the optical STEM signal detection employed in our setup. The q4STEM thickness determination method relies on MC simulations of the scattering process of transmitted electrons and currently depends on parametric approximations of material composition and uniformity. Based on theoretical considerations, these approximations may lead to a thickness over- or underestimation of ∼2%–3% for vitreous cellular material.

Alternative to an LM coincident with the FIB, an *in situ* reflected LM might also be used for thickness determination through the geometry. Although this would require stage moves between the LM and FIB, it can be implemented through software automation in commercially available systems.^15^^,^^16^^,^^36^

## Resource availability

### Lead contact

Further information and requests for resources should be directed to and will be fulfilled by the lead contact, Jacob P. Hoogenboom (j.p.hoogenboom@tudelft.nl).

### Materials availability

This study did not generate new unique reagents.

### Data and code availability

• All original data used in this study have been deposited at 4TU.ResearchData and are publicly available as of the date of publication. The DOI is listed in the key resources table.
• Data analysis scripts used in this study have been deposited at 4TU.ResearchData and are publicly available as of the date of publication. The DOI is listed in the key resources table.
• Any additional information required to reanalyze the data reported in this paper is available from the lead contact upon request.

## Acknowledgments

We thank Ernest B. van der Wee for the useful discussions and extend our gratitude to Joyce van Loenhout and Leanid Kresik for providing cellular samples used during optimization of automated FIB milling. We are thankful for the helpful discussions on the cryo-TEM workflow with the groups of C. Genoud and H. Stahlberg during J.P.H.’s research stay at UNIL-EPFL. This work was financially supported by NWO-TTW project no. 17152 to J.P.H. and the 10.13039/501100000781European Research Council (ERC-StG-852880) to A.J.J.

## Author contributions

A.J.J. and J.P.H. conceived and initialized the collaboration between their two research groups. First-principle q4STEM experiments were carried out by R.S. and extended to the cryogenic workflow by D.B.B. D.B.B. performed the experiments on thickness determination through the use of reflected light imaging. C.T. prepared the various cellular samples and, together with D.B.B., performed cryo-FIB milling. Cryo-ET experiments were done by C.T. and W.H.E. D.B.B. drafted the manuscript together with A.J.J. and J.P.H. All authors provided feedback and commented on the final version.

## Declaration of interests

Parts of this work are covered in NL patent 2032641 (R.S., J.P.H., A.J.J., and D.B.B.) and 2028497 (D.B.B.). D.B.B. is an employee of Delmic BV. J.P.H. has a financial interest in Delmic BV.

## STAR★Methods

### Key resources table

**Table:** 

REAGENT or RESOURCE	SOURCE	IDENTIFIER
**Biological samples**		

HeLa cell line stably expressing mRFP-GFP-LC3B	Gift from Prof. Fulvio Reggiori	University of Aarhus, Denmark

**Deposited data**		

All data reported in this paper are found at	4TU.ResearchData	10.4121/aeb7cf.17-1b4a-4f09-a2fb-88d7875c672d

**Software and algorithms**		

AreTomo	Zheng et al.^37^	N/A
ChimeraX	Goddard et al.^38^	N/A
eC-CLEM	Paul-Gilloteaux et al.^33^	https://icy.bioimageanalysis.org/plugin/ec-clem/
EMAN2	Chen et al.^39^	N/A
Icy	De Chaumont et al.^40^	https://icy.bioimageanalysis.org/
ImageJ	Schneider et al.^41^	https://imagej.net/ij/index.html
Inkscape	N/A	https://inkscape.org/
Python analysis scripts	N/A	N/A
reported in this paper,	N/A	N/A
are found at	4TU.ResearchData	10.4121/aeb7cf.17-1b4a-4f09-a2fb-88d7875c672d
Odemis	Piel et al.^27^	https://www.delmic.com/

### Experimental model and study participant details

HeLa cells (originally female) were cultured in Roswell Park Memorial Institute 1640 Medium supplemented with 10% Fetal bovin serum at 37 °C with 5% ${\text{CO}}_{2}$. With the cells dispensed onto a grid, they were starved for 2 h using Hanks’ Balanced Salt Solution. The cell line has not been authenticated as they were gifted to our lab.

### Method details

#### Cell culture

Briefly, HeLa cells stably expressing mRFP-GFP-LC3B were cultured in Roswell Park Memorial Institute 1640 Medium (RPMI, Gibco) supplemented with 10% Fetal bovin serum (FBS) at 37 °C with 5% ${\text{CO}}_{2}$. Glow-discharged carbon-coated gold mesh grids (QF 2/2 AU 200, Quantifoil) were placed on 3D printed holder (see^42^) and cells were gently dispensed onto it to reach 40 to 50% cells confluency. Subsequently, cells were starved for 2 h using Hanks’ Balanced Salt Solution (HBSS, Gibco). For cryo-protection prior to plunge freezing we adapted a protocol by^43^: After removal of HBSS, cells were submerged in medium supplemented with cryoprotectant at increasing concentrations (RPMI/10% FBS with DMSO/Glycerol 1.75%, DMSO/Glycerol 2.5%, DMSO/Glycerol 3.5% and DMSO/Glycerol 7%). Grids were removed from the holder, 2 μL of the final cryo-protectant solution was added on the back side before placing the grid on a Leica GP2 vitrification robot. Grids were blotted from the back for 12 s in the chamber maintained at 98% humidity and finally plunged into liquid ethane. Grids were clipped in Autogrid cartridges with milling notch before transfer to the FIB/SEM holder.

#### Microcrystal formation

Hen egg-white lysozyme microcrystals were grown using room temperature batch crystallization. Briefly, hen egg-white lysozyme was dissolved to 50 mg/mL in 20 mm sodium acetate pH 4.6. 100 μL of this solution were then mixed in a microcentrifuge tube with 250–400 μ L of precipitant solution consisting of 18% (w/v) NaCl, 6% (w/v) PEG 6000, 0.1 m sodium acetate pH 3.0. Lysozyme microcrystals formed immediately as evidenced by the turbid solution. Samples using different protein to precipitant ratios were screened using negative staining TEM to optimize conditions for homogeneous microcrystal formation with dimensions smaller than 5 μm. A 1:4 (v/v) ratio of protein to precipitant solution was found to yield the most homogeneous microcrystal populations. For vitrification, 3 μL of freshly prepared microcrystal slurry was applied on the carbon side of a glow-discharged QF R2/1 200 mesh grid (Quantifoil) kept at room temperature and 98% humidity on a Leica GP2 vitrification robot, blotted from the carbon side for 12 s and flash-frozen in liquid ethane. Prior to loading in the FIB-SEM, the grids were mounted in FIB-compatible AutoGrid cartridges and secured with a c-clip (Thermo Fisher Scientific).

#### Lamella fabrication

Lamellae were fabricated using the focused ion beam on a Helios Nanolab 650 (Thermo Fisher Scientific) operating at 30 kV. Rough milling started at 2.5 nA and when decreasing lamella thickness the current decreased to 0.79, 0.23 and 0.08 nA.

#### Energy-filtered TEM

Thickness determination in EFTEM was done on a JEOL JEM3200-FSC operated at 300 kV by acquiring unfiltered and zero-loss filtered images of the lamella at 2500 × magnification (3.59 nm per pixel) on a Gatan K2 Summit direct electron detector either with- or without a 20 eV slit inserted below the omega filter. Montages of the lamella were generated with PySerialEM.^44^ Aligned montages were used to compute $I/I_{0}$ by dividing the pixel values of the zero-loss image by those of the unfiltered image. A thickness map was finally obtained by mapping 320 nm $\cdot \ln\left(I_{0}/I\right)$ onto the lamella montage, where a literature value of 320 nm for the inelastic mean free path of 300 kV electrons in vitreous ice was used.^8^

#### Cryo-electron tomography

Lamellae were prepared as described above and stored in liquid nitrogen prior to imaging. Tilt series was acquired on a JEOL JEM3200-FSC operated at 300 kV with a bidirectional tilt scheme starting from 0 to −60° and subsequently from 2° to 60°, with a 2° tilt increment. At a magnification of 6000 ×, the pixel size was 6.3°A and a defocus of −3 μm was used. The tilt series was aligned using patch tracking and reconstructed using weighted back-projection as implemented in AreTomo.^37^ Segmentation was done in EMAN2 using a machine learning approach.^39^ Correlation between LM images and micrographs was performed using the ecCLEM plugin in ICY software^45^; tomogram and volume visualisation was done in ChimeraX.^38^

#### Scintillator selection for low temperatures

In Table S1, YAG is listed with other scintillator candidates are listed having a specified optical emission spectrum shifted toward lower wavelengths. LuAP was ordered (0.17 mm thick, 0.1 atomic percent cerium doped LuAlO_3_, single crystal, (110) oriented, Surface Preparation Laboratory B.V.) and several 3.4 mm diameter discs were laser cut. To avoid charging effects during electron beam exposure, the scintillator was coated with a 40 to 50 nm thick carbon film by means of thermal evaporation deposition. With an approximate specimen to scintillator distance of 300 μm, a maximum scattering angle of $\theta_{u}=$ 218 mrad can be measured.^23^ We have characterized how the electron induced scintillation yield changes as a function of temperature. A fiber coupled spectrograph (Princeton Instruments Acton SP2156 with a PyLoN:100BR Excelon camera) is connected on the excitation lights source (LS) input on the optics module, and the dichroic filter (DF) is replaced with a 50/50 beamsplitter (for details on the optical components see^17^). This allows collection of scintillator emission spectra whilst also imaging the scintillation spot on the camera. A primary electron beam energy of 5 keV is set along with a 0.1 nA beam current. Emission spectra are recorded whilst cooling down from 300 to 110 K as shown in Figure S1B, top. No spectral shift is recorded, but the maximum intensity decreases by a factor of 2.5 (Figure S1B, bottom). LuAP, given its orthorhombic crystal structure, introduces birefringence into the optical system, which is filtered out by adding a polarizing beamsplitter (PBS) in front of the camera (water-cooled Andor Zyla 4.2 PLUS). Depending on the orientation of the PBS either the parasitic pattern originating from the scintillator birefringence or the electron induced scintillation spot can be imaged, see Figure S1C. Without PBS present in the optical path, both are seen simultaneously (top).

### Quantification and statistical analysis

The thickness uniformity reported in the main text is through one Standard Deviation Figure 6D (bottom right).

## Supplemental information

Document S1. Figures S1–S5, Table S1, and supplemental references

Document S2. Article plus supplemental information
