# Supplementary material for: Thickness- and quality-controlled fabrication of fluorescence-targeted frozen-hydrated lamellae
Source: Cell Rep Methods. 2025 Mar 24;5(3):101004. doi: 10.1016/j.crmeth.2025.101004 (PMC12049727; doi:10.1016/j.crmeth.2025.101004)
Supplement: Document S1. Figures S1–S5, Table S1, and supplemental references [file mmc1.pdf]

Cell Reports Methods, Volume *5*

Supplemental information

Thickness- and quality-controlled fabrication of fluorescence-targeted frozen-hydrated lamellae

Daan B. Boltje, Radim Skoupý, Clémence Taisne, Wiel H. Evers, Arjen J. Jakobi, and Jacob P. Hoogenboom

The following is included in the supplemental information:
Figures S.1 to S.5
Table S.1

Table S.1: **Overview of scintillator candidates, related to STAR Methods.**

Scint.	Cryst. struct.	λ_{ab} [nm]	λ_{em}^{max} [nm]	BF	Ref
YAG	C	460, 340	550	No	[S1]
YAP	OR	300	370	Yes	[S1,S2]
LYSO	MC	250, 360	425	Yes	[S1,S3,S4]
LuAP	OR	300	365	Yes	[S1,S5–S7]

C, OR and MC refer to cubic, orthorhombic or monoclinic,
BF indicates birefringence.

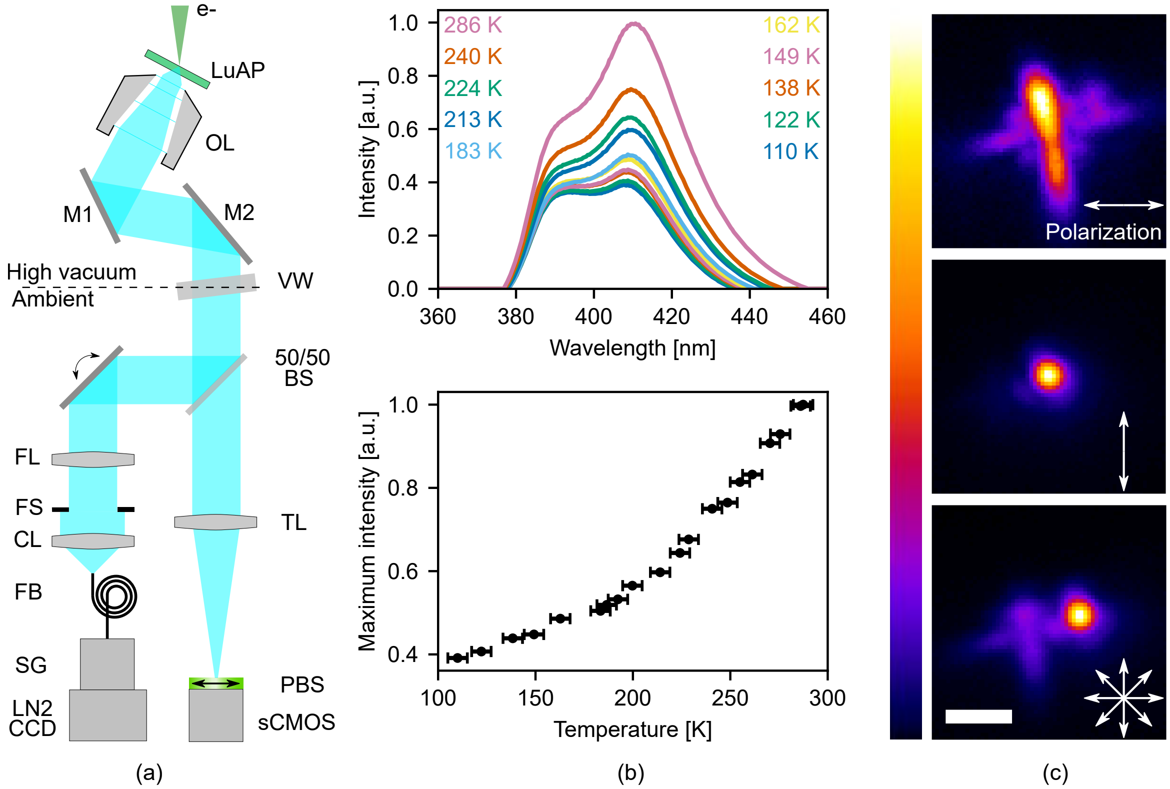

Figure S.1: **Optical properties of a LuAP scintillator under electron irradiation and the layout of the optical path, related to STAR Methods.** **(a)** Overview of the optical path denoting relevant components. The LuAP scintillator is irradiated with $5\,\mathrm{keV}$, $100\,\mathrm{pA}$ electons. The scintillation light is collected by the objective lens (OL) and passed to the 50/50 beam splitter (BS), where it is divided between the excitation- and emission arms. This allows part of the light to be passed to the tube lens (TL) and focussed onto the camera (sCMOS) to form an image. A polarizing beam splitter (PBS) is added in front of the camera. The fiber coupled light source is replaced with a spectograph (SG) fitted with a liquid nitrogen cooled CCD camera. **(b)** Electron induced scintillaton emission spectra as a function of scintillator temperature (top). Peak positions do not shift, but the scintillation yield decreases. Peak maxima are plotted against temperature (bottom). The scintillation yield decreases with a factor of 2.5 going from 286 to $110\,\mathrm{K}$. Horizontal errorbars of $5\,\mathrm{K}$. **(c)** Scintillator birefringence is visualized by imaging the electron induced scintillation spot with the optical system. Without PBS present two spots are present (top) and the parasitic spot (middle) can be filtered out (bottom) depending on the PBS orientation. Scale bars: **(c)** $2\,\mathrm{\mu m}$.

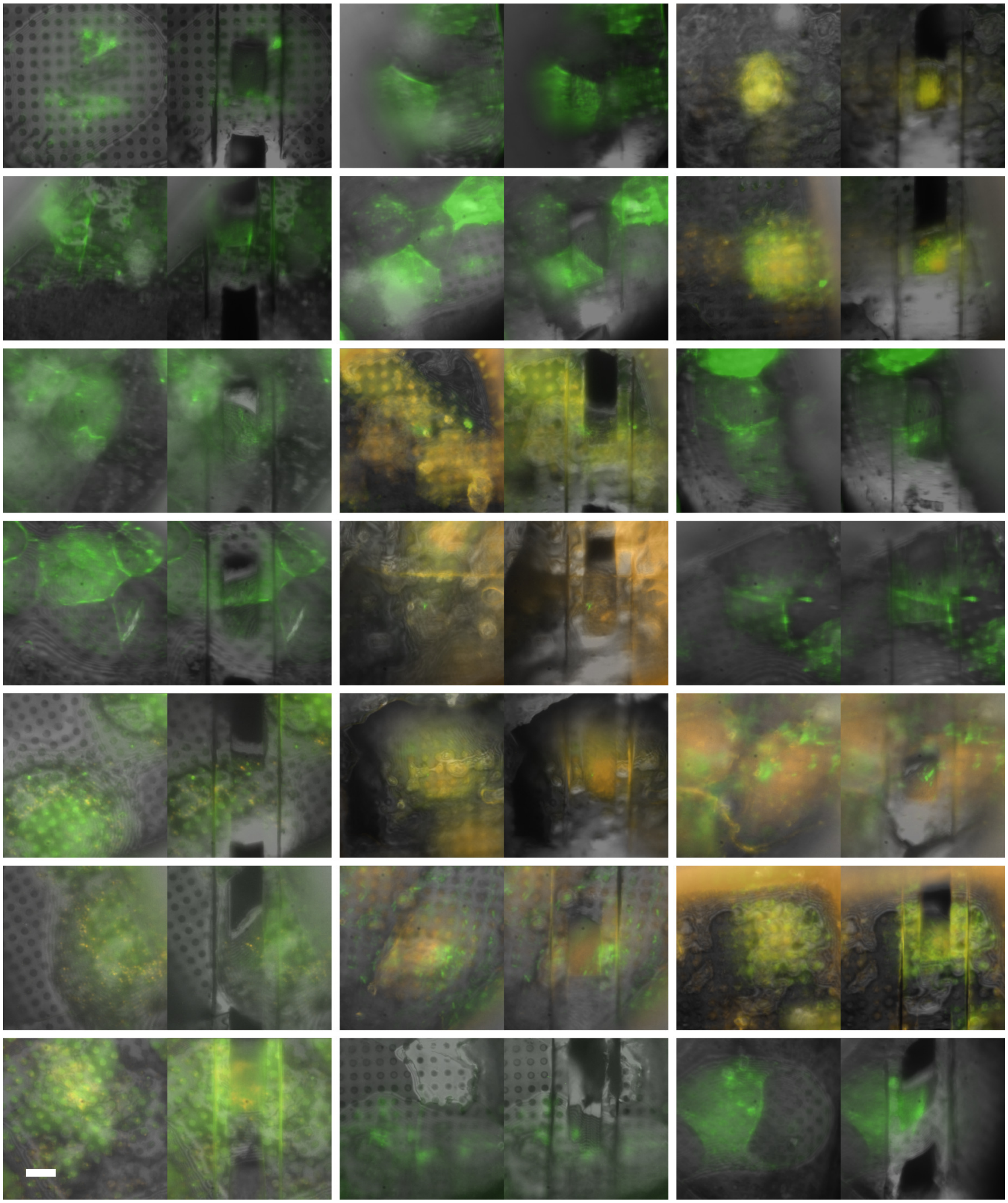

Figure S.2: **Fluorescent (colors) overlaid on reflected light (grayscale) image pairs as obtained by automated milling, related to Figure 2.** For each set, the left image is acquired prior to rough milling and the right image after. Scale bar $10\,\mu m$.

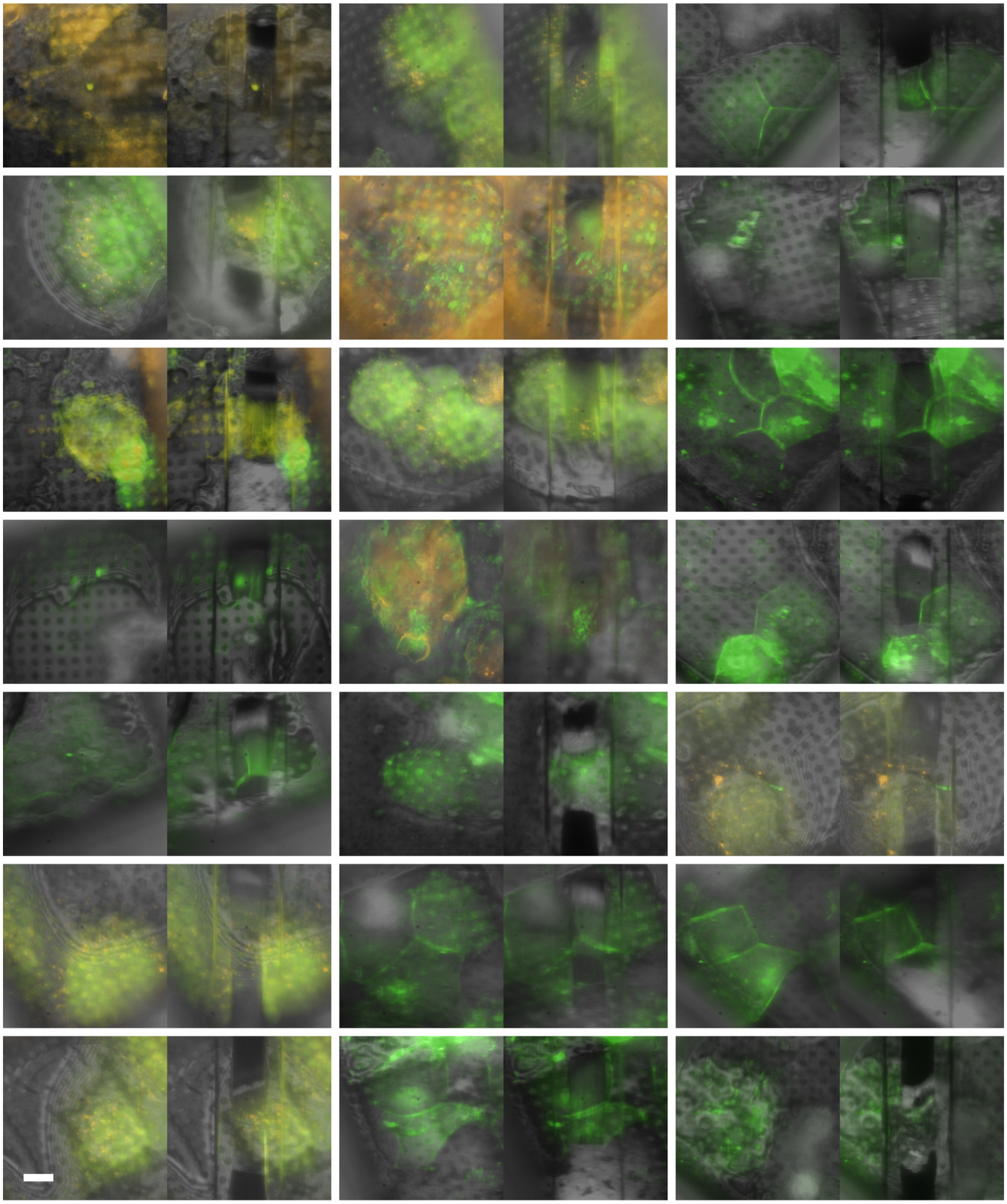

Figure S.3: **Fluorescent (colors) overlaid on reflected light (grayscale) image pairs as obtained by automated milling, related to Figure 2.** For each set, the left image is acquired prior to rough milling and the right image after. Scale bar $10\,\mu\mathrm{m}$.

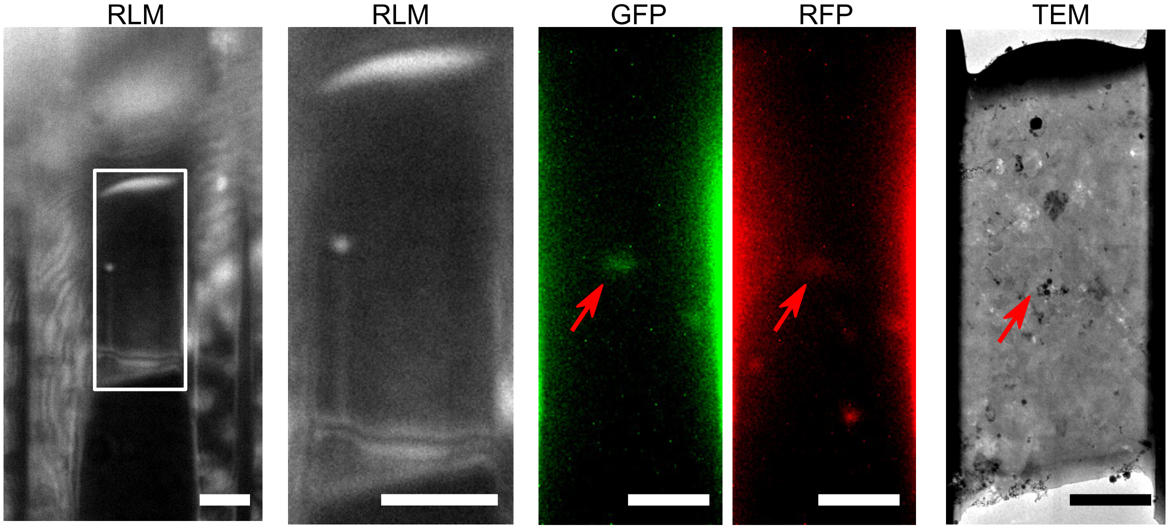

(a) (b) (c) (d)

Figure S.4: **Fluorescence targeted lamella as fabricated for q4STEM thickness determination and verification with energy filtered transmision electron microscopy (EFTEM), related to Figure 3.** A red fluorescent protein (RFP)-GFP tandem fluorescent-tagged LC3 (mRFP-GFP-LC3) single molecule-based probe is used that can monitor the autophagosome maturation process[S8]. **(a)** lamella as imaged with reflected light microscopy (RLM) after thinning with the FIB, and in **(b)** zoom from white rectangle. **(c)** Separated fluorescence microscopy channels, imaged after polishing (white rectangle from **(b)**), with target present (red arrows). From left to right: GFP emission ($525\,\mathrm{nm}$ in green), and RFP emission ($607\,\mathrm{nm}$ in orange). **(d)** Overview image as acquired in the transmission electron microscope (TEM). All scale bars: $5\,\mathrm{\mu m}$.

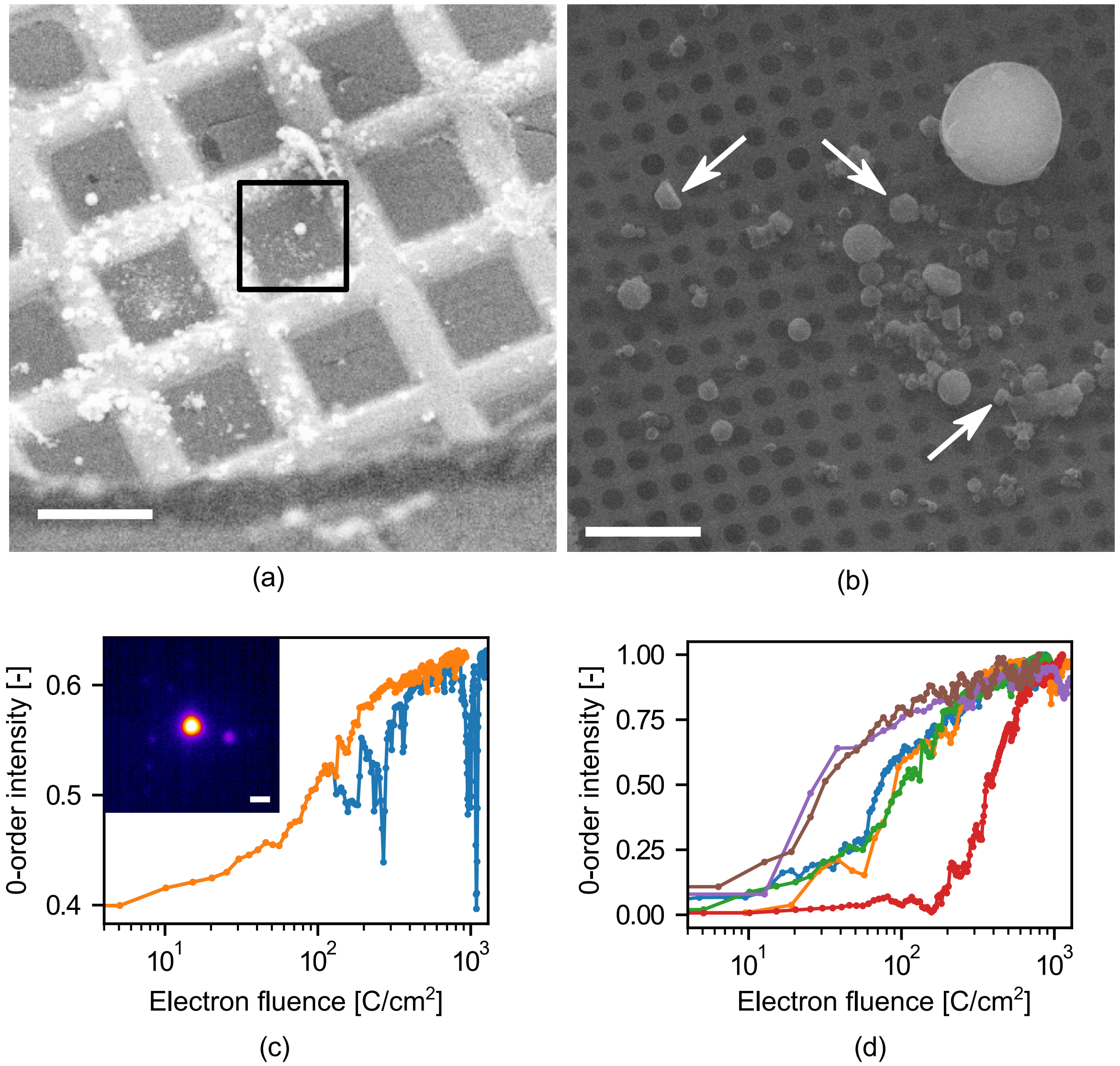

Figure S.5: **Assessment of radiolytic damage by q4STEM measurements, related to Figure 3.** **(a)** Overview image of the electron microscope (EM) grid containing small protein crystals. **(b)** The area as indicated in (a, black rectangle) shown at a larger magnification. White arrows denote the crystals which showed (partial) diffraction patterns. **(c)** With the optical focus set to the scintillator, the electron beam was moved over each crystal in search of a (partial) diffraction pattern (example shown in inset). Once found, a time-lapse acquisition was started, from which the 0-order intensity is plotted against electron fluence. The discontinuities seen in the blue trace originate from ~ 2 to $4\,\mathrm{nm}$ peak-to-peak sample vibrations, causing slightly different parts of the crystal to be exposed[S9]. By enforcing a monotonic increase, we filter and remove these discontinuities (orange trace). **(d)** Filtered 0-order intensity against electron fluence for different crystals, as indicated by the white arrows in (b). The electron fluence is determined through the estimated beam diameter $(10\,\mathrm{nm})$ and the set beam current $(25$ to $100\,\mathrm{pA})$. Scale bars: **(a)** $100\,\mu\mathrm{m}$, **(b)** $10\,\mu\mathrm{m}$, **(c, inset)** $6\,\mu\mathrm{m}$ or $20\,\mathrm{mrad}$.

References

S1. Crytur. *Scintillator physical properties*, 2023. https://www.crytur.com/materials/yagce/ (Retrieved 14-09-2023).

S2. Samuel B Donnald, Richard Williams, Charles L Melcher, Fang Meng, Merry Koschan, Stephan Friedrich, Jesse A Johnson, and Jason P Hayward. Correlation of nonproportionality and scintillation properties with cerium concentration in yalo 3: Ce. *IEEE Transactions on Nuclear Science*, 65(5):1218–1225, 2018.

S3. S Blahuta, A Bessiere, B Viana, P Dorenbos, and V Ouspenski. Evidence and consequences of Ce^{4+} in lyso: Ce, ca and lyso: Ce, mg single crystals for medical imaging applications. *IEEE Transactions on Nuclear Science*, 60(4):3134–3141, 2013.

S4. Ludivine Pidol, Andrée Kahn-Harari, Bruno Viana, Eric Virey, Bernard Ferrand, Pieter Dorenbos, Johan TM de Haas, and Carel WE van Eijk. High efficiency of lutetium silicate scintillators, ce-doped lps, and lyso crystals. *IEEE Transactions on Nuclear Science*, 51(3):1084–1087, 2004.

S5. Winicjusz Drozdowski, Andrzej J Wojtowicz, Tadeusz Łukasiewicz, and Jarosław Kisielewski. Scintillation properties of luap and luyap crystals activated with cerium and molybdenum. *Nuclear Instruments and Methods in Physics Research Section A: Accelerators, Spectrometers, Detectors and Associated Equipment*, 562(1):254–261, 2006.

S6. A Lempicki and J Glodo. Ce-doped scintillators: Lso and luap. *Nuclear Instruments and Methods in Physics Research Section A: Accelerators, Spectrometers, Detectors and Associated Equipment*, 416(2-3):333–344, 1998.

S7. Surface preparation laboratory. *Premier supplier of single crystal surfaces - SPL*, 2023. https://www.spl.eu/ (Retrieved 14-09-2023).

S8. Shunsuke Kimura, Takeshi Noda, and Tamotsu Yoshimori. Dissection of the autophagosome maturation process by a novel reporter protein, tandem fluorescent-tagged lc3. *Autophagy*, 3(5):452–460, 2007.

S9. Daan B Boltje, Jacob P Hoogenboom, Arjen J Jakobi, Grant J Jensen, Caspar TH Jonker, Max J Kaag, Abraham J Koster, Mart GF Last, Cecilia de Agrela Pinto, Jürgen M Plitzko, et al. A cryogenic, coincident fluorescence, electron, and ion beam microscope. *Elife*, 11:e82891, 2022.
